# Exploring Underrecognized Risk Factors and Diagnosis of Postpartum Eclampsia: A Case Report

**DOI:** 10.7759/cureus.110425

**Published:** 2026-06-07

**Authors:** Munazza Shaikh, Sri Hari Babu Sunkari, Ajay A

**Affiliations:** 1 Department of Trauma and Emergency, All India Institute of Medical Sciences, Nagpur, Nagpur, IND

**Keywords:** case report, hypertension of pregnancy, hypothyroidism, late postpartum eclampsia, lscs, magnesium sulfate, no prior hypertension, postoperative day 7, postpartum seizures

## Abstract

Eclampsia occurring beyond 48 hours postpartum is termed "late postpartum eclampsia" (LPPE), which is a clinically challenging condition. It has an occurrence of approximately 15% of all eclampsia cases and often presents in women with no documented antenatal hypertension. We report a case of LPPE manifesting on postoperative day (POD) 7 following elective lower-segment cesarean section (LSCS) in a primigravida with pre-existing hypothyroidism in the complete absence of hypertensive recordings prior to and during the antenatal or immediate postoperative period. This case adds to the limited literature by highlighting potential risk factors and the need for a high index of suspicion. A 21-year-old primigravida woman (P1L1), POD 7 after elective LSCS for cephalopelvic disproportion, presented to the emergency department with a six-hour prodrome of severe occipital headache, blurring of vision, and two witnessed generalized tonic-clonic seizures. She had a background history of hypothyroidism managed with levothyroxine 25 µg once daily with no recorded hypertension prior to or during antenatal visits or during her initial postoperative hospital stay. Vital parameters recorded during the post-ictal phase were BP 140/92 mmHg, pulse 90 per minute, saturation 98% on room air, and random blood sugar level 92 mg/dl. She was administered intravenous levetiracetam and shifted for a CT brain, which showed no abnormality. Investigations revealed a spot urine protein-to-creatinine ratio of 308 mg/g with normal hepatic and renal parameters. She was diagnosed with LPPE based on new-onset hypertension, significant proteinuria, and neurological involvement. She was later managed with intravenous magnesium sulfate (MgSO₄) and antihypertensive therapy. After clinical stabilization, she was discharged on POD 13. This case underscores that LPPE can occur as late as POD 7 in the absence of documented hypertension, which makes anticipatory management difficult. It also highlights the need for structured postpartum surveillance protocols and explicit patient and family education regarding warning symptoms.

## Introduction

Eclampsia is defined as the occurrence of one or more grand mal seizures superimposed on a hypertensive disorder of pregnancy. It remains a leading cause of maternal morbidity and mortality globally, with an estimated incidence of 1.6-10 per 10,000 deliveries in high-income countries and significantly higher rates in low- and middle-income settings [1].

Postpartum eclampsia is defined as eclampsia occurring in the postpartum period. Immediate postpartum eclampsia refers to seizures arising in the first 48 hours postpartum. Late postpartum eclampsia (LPPE) traditionally encompasses seizures arising beyond the first 48 hours postpartum [2,3]. LPPE is a diagnostically challenging entity as it occurs at a time when obstetric vigilance may have diminished, patients have often been discharged during the particular time frame, and the clinical presentation mimics other neurological emergencies, including stroke, subarachnoid hemorrhage, hypertensive encephalopathy, new-onset epilepsy, reversible cerebral vasoconstriction syndrome (RCVS), or cerebral venous sinus thrombosis (CVST).

Studies suggest that up to 21% of women with postpartum eclampsia have no identifiable hypertension during the antenatal or intrapartum period. The pathophysiology of such cases likely involves a combination of endothelial dysfunction, cerebrovascular dysregulation, and postpartum hormonal flux. The potential modulating role of thyroid dysfunction in hypertensive disorders of pregnancy is an area of growing interest, as hypothyroidism has been associated with endothelial dysfunction, dyslipidemia, and hyperhomocysteinemia, all of which may lower the threshold for cerebrovascular events [4]. Lower-segment cesarean section (LSCS) can also be a contributory risk factor for LPPE.

## Case presentation

Patient information

A 21-year-old primigravida woman (P1L1) presented to the emergency department with headache and blurring of vision followed by two episodes of seizures. She had undergone elective LSCS seven days prior at a secondary care facility for CPD, which was uneventful, and she was discharged on POD 4 in satisfactory condition.

Past medical, obstetric, and drug history

The patient is a P1L1 with a significant medical history of hypothyroidism, diagnosed five years ago and currently managed with 25 µg of levothyroxine sodium once daily. Her obstetric and family histories are unremarkable for hypertensive disorders of pregnancy, specifically noting the absence of gestational hypertension, pre-eclampsia, or superimposed hypertension during her prior antenatal period. Prior to the current pregnancy, she reported regular menstrual cycles with an average flow. She maintains healthy lifestyle habits with no history of addictions and reports normal sleep, appetite, and bowel and bladder functions.

Clinical findings on admission

At presentation to the emergency department, the patient was brought in in the post-ictal phase. She later recovered and became conscious and oriented to time, place, and person. Physical examination findings are mentioned in Table 1.

**Table 1:** Clinical examination findings on examination SpO₂: peripheral oxygen saturation, GCS: Glasgow Coma Scale, CNS: central nervous system, LSCS: lower segment cesarean section, BP: blood pressure

Parameter	Finding
Temperature	Afebrile
Heart rate	92 bpm, regular
BP	140/92 mmHg
SpO₂	98% on room air
GCS/orientation	E4V5M6, fully conscious and oriented to time, place, and person
Pallor/Icterus	Absent
Pedal edema	Absent on admission
Cardiovascular	S1 and S2 heard, no murmurs
Respiratory	Bilateral air entry clear, no added sounds
CNS	Conscious and oriented; deep tendon reflexes: within normal limits, no focal neurological deficits
Abdomen	Uterus well-contracted, LSCS suture line healthy, no tenderness

Diagnostic assessment

A CT brain was done, which was reported as normal and with no evidence of cerebral edema, hemorrhage, or infarction. Ultrasound of the kidneys showed no structural abnormality, hydronephrosis, or renal parenchymal disease. Fundoscopy was performed, showing no papilledema or hypertensive retinal changes. An MRI brain with angiography and venography was done, which was reported as normal and showed no evidence of posterior reversible encephalopathy syndrome (PRES), cerebral edema, hemorrhage, or infarction. Laboratory parameters and values are enumerated in Table 2.

**Table 2:** Summary of laboratory investigations HELLP: hemolysis, elevated liver enzymes, and low platelet count, HIV: human immunodeficiency virus, HBsAg: hepatitis B surface antigen, HCV: hepatitis C virus, TSH: thyroid-stimulating hormone, LDH: lactate dehydrogenase

Investigation	Result	Reference range	Interpretation
Hemoglobin	12.4 g/dL	12-16 g/dL	Normal
Platelet count	348 × 10³/µL	150-450 × 10³/µL	Normal (rules out thrombocytopenia of HELLP)
White cell count	12.63 × 10³/µL	4-11 × 10³/µL	Mildly elevated (post-surgical/post-ictal)
Liver function tests	Within normal limits	-	No hepatic involvement; HELLP criteria not met
Renal function tests	Within normal limits	-	No acute kidney injury
HIV	Negative	Negative	Infectious etiology excluded
HBsAg	Negative	Negative	Infectious etiology excluded
HCV	Negative	Negative	Infectious etiology excluded
Spot urine protein-to-creatinine ratio	308 mg/g (0.38 mg/mg)	<300 mg/g	Proteinuria present (consistent with pre-eclampsia spectrum)
T3	0.77 ng/ml	0.60-1.81 ng/ml	Hypothyroidism background, lab values within normal limits
T4	12.3 micro g/dl	5-13 micro g/dl	Hypothyroidism background, lab values within normal limits
TSH	1.32 micro IU/ml	0.5-5 micro IU/ml	Hypothyroidism background, Lab values within normal limits
LDH	582 U/l	140-280 U/l	Not elevated more than three times the upper limit of normal

Diagnostic conclusion

**Figure 1:**
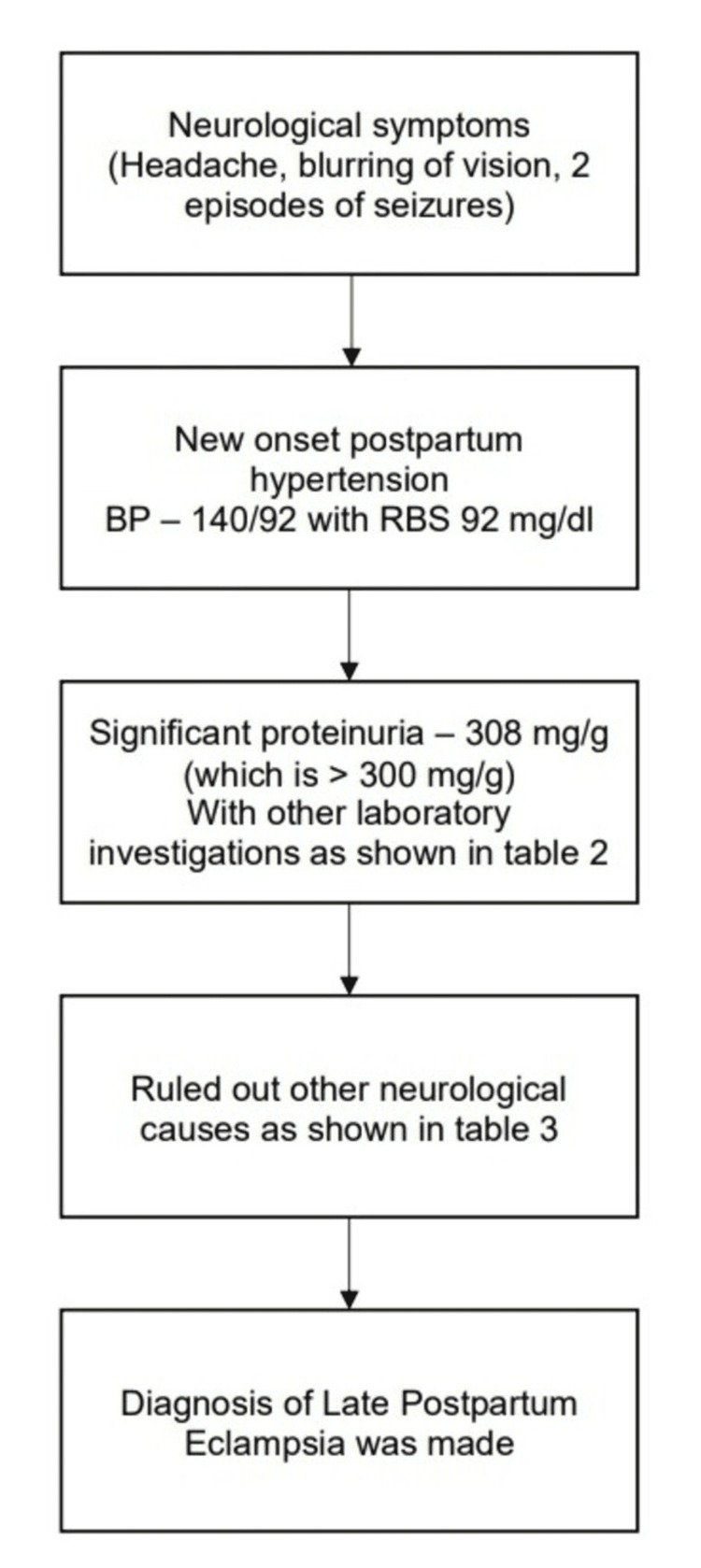
Stepwise approach for differentiating LPPE from other causes of seizures BP: blood pressure, RBS: random blood sugar, LPPE: late postpartum eclampsia

The clinical picture (two episodes of generalized seizures in the postpartum period with new-onset hypertension (140/92 mmHg), significant proteinuria, no prior hypertensive history, and absence of alternative neurological etiology as shown in Table 3) is consistent with a diagnosis of LPPE as shown in Figure 1. HELLP syndrome was excluded (normal platelets, LFT, and hemoglobin). Hypothyroidism was a pre-existing comorbidity. MRI negativity for PRES is acknowledged as a limitation, though PRES can be radiologically subtle in the early phase.

**Table 3:** Differential diagnosis of postpartum eclampsia DWI: diffusion-weighted imaging, NCCT: non-contrast computed tomography, MRA: magnetic resonance angiography, MRV: magnetic resonance venography, RCVS: reversible cerebral vasoconstriction syndrome, CVST: cerebral venous sinus thrombosis, MRI: magnetic resonance imaging, CT: computed tomography

Differential diagnosis	Key CT/MRI findings	How it was ruled out
Acute ischemic stroke	MRI (DWI): focal areas of restricted diffusion corresponding to a specific arterial territory	Absence of restricted diffusion on DWI and lack of focal arterial territory infarction
Acute intraparenchymal bleed	NCCT: acute intraparenchymal bleed seen as hyperdense on CT	NCCT showed no evidence of hemorrhage
RCVS	MRA: characteristic "string of beads" appearance	MRA showed normal caliber vessels without multifocal segmental vasoconstriction
CVST	MRV: filling defects in the dural venous sinuses	MRV confirmed patency of all major dural venous sinuses
Subdural hemorrhage	NCCT: acute intraparenchymal or subarachnoid blood seen as hyperdense on CT	NCCT showed no evidence of hemorrhage
Subarachnoid hemorrhage	NCCT: acute subarachnoid blood seen as hyperdense on CT	NCCT showed no evidence of hemorrhage
Meningitis/encephalitis	MRI with contrast shows leptomeningeal enhancement	MRI did not show any areas of enhancement
Post-dural puncture headache	MRI brain shows effacement of basilar cisterns	MRI showed no effacement

Therapeutic interventions

For seizure control and prophylaxis, the patient was initiated on the standard Zuspan regimen of intravenous magnesium sulfate (MgSO₄), beginning with a 4 g loading dose over 15-20 minutes, followed by a maintenance infusion of 1 g/hour for 24-48 hours. Throughout this administration, clinical safety was ensured by strictly monitoring deep tendon reflexes, respiratory rate, and urine output. While a 1000 mg dose of intravenous Levetiracetam was initially commenced as an empirical antiepileptic measure prior to neuroimaging, it was subsequently discontinued after a CT brain scan returned normal results. The clinical team determined that the seizures were consistent with postpartum eclampsia rather than primary epilepsy, prioritizing MgSO₄ as the definitive treatment.

Hypertension was managed with tablet nifedipine, administered as needed to maintain a target BP of less than 140/90 mmHg. To ensure systemic stability, continuous supportive measures were implemented, including the rigorous monitoring of vital signs such as heart rate, SpO₂, respiratory rate, and hourly urine output.

Follow-up and outcome

Following clinical stabilization with MgSO₄ and antihypertensives, the patient was admitted to the intensive care unit under obstetrics and gynecology. Later, the patient was shifted for a brain MRI, which was reported as normal, ruling out the possibility of PRES. The patient's neurological status normalized, and no further seizures were recorded during the hospital stay. During hospitalization, antihypertensive therapy was titrated and supportive care was provided. The patient was shifted to the ward and was discharged on POD 13 (day 6 of hospitalization) in fair general condition. At the time of discharge, specific advice was given regarding BP monitoring, immediate return to emergency if symptoms recur, strict continuation of medications, and follow-up within 72 hours. The timeline of events is illustrated in Table 4.

**Table 4:** Chronological summary of clinical events LSCS: lower segment cesarean section, CPD: cephalopelvic disproportion, POD: postoperative day, NCCT: non-contrast computed tomography, MgSO₄: magnesium sulfate, ICU: intensive care unit

Time/day	Event
17 Dec 2024	Elective LSCS for CPD (POD 0); male neonate, 3.1 kg, full-term. Intraoperative course uneventful
21 Dec 2024	POD 4: Discharged home in stable condition. No hypertension documented during admission
24 Dec, 01:30	POD 7: Onset of severe occipital headache and vomiting
24 Dec, 05:30	Blurring of vision, worsening headache
24 Dec, 07:30	First generalized tonic-clonic seizure at home followed by loss of consciousness
24 Dec, 09:20	Second generalized tonic-clonic seizure on arrival to tertiary care hospital. Empirical anti-epileptics administered and shifted for NCCT brain
24 Dec, 13:19	MgSO₄ loading dose administered and shifted to ICU
24 Dec, 14:45	MgSO₄ maintenance commenced and shifted for MRI brain
POD 8-10	BP monitoring, investigations, neurology and medicine consultations. Levetiracetam initiated then discontinued per neurology advice. Antihypertensives (nifedipine) commenced
30 Dec 2024	Disposition - POD 13: discharged in a stable condition

## Discussion

Epidemiology and definition of LPPE

Postpartum eclampsia represents a substantial proportion of all eclampsia cases. The traditional 48-hour cutoff for classifying eclampsia as late is well established in the literature, with documented cases occurring up to six weeks postpartum [2]. In a landmark analysis by Lubarsky et al., LPPE (>48 hours) accounted for approximately 26% of all postpartum eclampsia cases, with a mean onset of 5.1 days postpartum, closely mirroring the present case [3]. Sibai reported that postpartum eclampsia constitutes 44% of all eclampsia cases in a large prospective series, and a significant minority occur beyond 48 hours [5]. Our patient developed eclampsia on POD 7, placing her at the outer limit but within the biologically plausible window. This has critical clinical implications, as the patient had been discharged home on POD 4 without any documented hypertension, and seizures developed after a 6-hour prodrome of headache and visual symptoms in the community. This underscores the inadequacy of postpartum discharge without structured BP monitoring protocols and explicit symptom counseling.

Eclampsia in the absence of prior hypertension

One of the most challenging aspects of this case is the complete absence of hypertension at any antenatal visit or during the initial LSCS hospitalization. Douglas and Redman (1994), in a UK population-based study (n = 383), found that 22% of women with eclampsia had no antecedent hypertension or proteinuria [6]. This phenomenon is attributed to the episodic and labile nature of vasospasm in the eclampsia spectrum, in which BP may not exceed diagnostic thresholds until the moment of acute decompensation. The World Health Organization definition of eclampsia does not mandate prior hypertension as a prerequisite [1]. Clinicians must therefore not be falsely reassured by a normotensive postpartum course. The presence of peripheral edema (noted by the patient for one month prior to admission), proteinuria, and prodromal neurological symptoms (headache and visual blurring) is sufficient to mandate emergency evaluation and MgSO₄ administration, even in the absence of documented hypertension [5].

Hypothyroidism as a possible modulating factor

This patient had a five-year history of hypothyroidism managed with a low-dose levothyroxine regimen (25 µg/day). Whether thyroid function was optimally controlled during pregnancy is unknown, as documentation was incomplete. This is clinically relevant for multiple reasons. Hypothyroidism is associated with endothelial dysfunction, elevated systemic vascular resistance, and impaired angiogenic signaling, which are pathophysiological pathways shared with pre-eclampsia. Subclinical hypothyroidism in pregnancy has been independently associated with an increased risk of gestational hypertension and pre-eclampsia in multiple meta-analyses [4,7]. The recommended levothyroxine dose in pregnancy is typically increased by 25-50% to accommodate the higher physiological demand for thyroid hormone; a dose of 25 µg/day is likely inadequate during gestation. Postpartum thyroiditis, occurring in up to 7-10% of women, can produce a transient thyrotoxic or hypothyroid phase in the weeks after delivery, potentially contributing to cardiovascular and neurological instability.

Neuroimaging and PRES considerations

The MRI brain was reported as normal in this case. Cerebral imaging suggests that cerebral abnormalities in eclampsia (mostly vasogenic edema) are similar to those found in hypertensive encephalopathy. However, cerebral imaging is not necessary for the diagnosis or management of most women with eclampsia [5]. PRES, the radiological correlate of hypertensive cerebral vasogenic edema frequently observed in eclampsia, was not demonstrated. However, MRI sensitivity for PRES can be limited in the hyperacute phase and may require repeat imaging 48-72 hours after onset [8]. Schwartz et al. described MRI abnormalities in approximately 75% of eclamptic women, predominantly in the parieto-occipital regions; however, radiological absence does not exclude the pathophysiological syndrome [9]. The clinical diagnosis of eclampsia remained secure in this case based on the clinical criteria, and the absence of MRI findings does not challenge it.

Antiepileptic choice: magnesium sulfate vs. levetiracetam

The Magpie Trial, an international RCT in 10,141 women with pre-eclampsia, established MgSO₄ as superior to phenytoin and diazepam for both the prevention and treatment of eclampsia (NNT = 63 to prevent one eclamptic seizure, NNT = 91 in the postpartum group) [10]. MgSO₄ remains the drug of choice for the management of eclampsia per WHO (2011), NICE (2019), ACOG, RCOG, and FIGO guidelines. In this case, levetiracetam was initiated empirically but subsequently discontinued after the diagnosis of LPPE. This decision is consistent with current evidence: levetiracetam has no established role in the management of eclampsia. It may divert attention from the primary therapeutic goals of MgSO₄ infusion and BP control. Emerging case series have explored levetiracetam as an adjunct in refractory eclampsia, but randomized evidence is lacking [11].

Clinical pearls

Never exclude eclampsia based on a normotensive antenatal record: Up to 22% of eclampsia cases have no prior documented hypertension. Postpartum eclampsia can occur beyond one week of delivery: the late postpartum period (up to six weeks) remains a vulnerability window, and public awareness is critical. The classical prodrome (headache and visual disturbance) is a medical emergency: any postpartum woman presenting with these symptoms requires immediate BP measurement, urinalysis, and assessment for seizure risk. MgSO₄ remains the cornerstone of management: Do not substitute with antiepileptic agents; neurological consultations should reinforce, not replace, obstetric management. Hypothyroid women require levothyroxine dose optimization during pregnancy: subtherapeutic dosing may contribute to endothelial and cardiovascular vulnerability in the peripartum period. MRI negativity does not exclude eclampsia or PRES: The diagnosis is fundamentally clinical; repeat imaging at 48-72 hours may be informative.

## Conclusions

This case of LPPE on POD 7 in a primigravida with hypothyroidism and no prior hypertensive history illustrates several underappreciated facets of this condition, such as the possibility of eclampsia well beyond the immediate postpartum period, the occurrence without antecedent hypertension, and the potential confounding role of comorbid hypothyroidism. Structured postpartum surveillance protocols, including home BP monitoring, explicit symptom education, and early community follow-up, are warranted for all women who have undergone cesarean section, particularly those with risk factors for pre-eclampsia spectrum disorder. A high index of clinical suspicion, prompt administration of MgSO₄, and meticulous BP control remain the pillars of management.
